# Understanding Nursing Turnover: The Case of Home Health Care

**DOI:** 10.1093/geroni/igaa057.114

**Published:** 2020-12-16

**Authors:** Joanne Spetz, Alon Bergman, Hummy Song, Amber Rose, Guy David

**Affiliations:** 1 University of California, San Francisco, San Francisco, California, United States; 2 University of Pennsylvania, Philadelphia, Pennsylvania, United States

## Abstract

Only a few studies of nursing turnover have examined post-acute home health care. This study examines factors that are associated with home health licensed nurse turnover using linked employee-level and patient-level data from one of the five largest home health companies in the US. The data include variables from human resources and payroll systems, visit logs, discharge records, physical and mental health assessments, care plans, and patient encounters and is organized at the employee-day level. We measured turnover using human resources data, including measures of voluntary and involuntary job separation, and from exit interviews that allow classification of whether turnover was associated with agency-related factors (e.g., pay, schedule, supervisor, coworkers) versus personal factors (e.g., family needs, relocation). In bivariate and multivariate analyses, explanatory variables included nurse demographics, patient population characteristics, and the degree to which nurses can delegate tasks to home care aides. We found a downward trend in turnover for licensed nurses between 2016 and 2019. Attrition in the first year was 34% for full-time nurses and 45% for part-time nurses, most of it occurring in the first 180 days of employment. The rate of voluntary turnover was nearly four times as great as involuntary turnover. We found that agency factors accounted for 26% of monthly turnover on average, while personal factors accounted for 74%. In states in which licensed nurses could delegate more tasks to home care aides, turnover rates were slightly higher than in states with little delegation.

